# TMEM16A ablation in cholinergic medial habenula neurons induces early-onset schizophrenia-like phenotypes in mice

**DOI:** 10.1186/s13041-025-01266-y

**Published:** 2026-01-12

**Authors:** Ajung Kim, Soomin Lee, Sangjoon Lee, Jeongyeon Kim, Heh-In Im, Jae-Young Park, Eun Mi Hwang

**Affiliations:** 1 Brain Science Institute, Korea Institute of Science and Technology (KIST), Seoul, 02792 Republic of Korea; 2 Department of Integrated Biomedical and Life Science, Graduate School, Korea University, Seoul, 02841 Republic of Korea; 3 Emotion, Cognition and Behavior Research Group, Korea Brain Research Institute, Daegu, 41062 Republic of Korea; 4 Division of Bio-Medical Science & Technology, KIST School, University of Science and Technology (UST), Seoul, 02792 Republic of Korea; 5 Division of Food Functionality Research, Korea Food Research Institute, Wanju, 55365 Republic of Korea

**Keywords:** Schizophrenia, Conditional knockout mice, TMEM16A, Medial habenula

## Abstract

Schizophrenia is a heterogeneous psychiatric disorder that remains inadequately treated with current therapies. Developing appropriate animal models that reflect the broad spectrum of schizophrenia symptoms is crucial for advancing our understanding of the disease and identifying effective treatments. However, existing animal models often have limitations in fully recapitulating the diverse symptomatology observed in humans. Previously, we reported that mice with conditional ablation of TMEM16A (ANO1) in cholinergic neurons of the medial habenula (ANO1 cKO) exhibit behavioral patterns indicative of anxiety, reduced social motivation, and anhedonia. In the present study, we found that these mice display schizophrenia-like phenotypes, including impaired prepulse inhibition (PPI), enhanced cocaine sensitivity, and reduced c-Fos expression in the medial prefrontal cortex (mPFC), a feature also observed in patients with schizophrenia. Moreover, ANO1 cKO mice exhibited elevated Drd2 expression in the ventral medial geniculate nucleus (MGv) and transcriptomic alterations overlapping with schizophrenia-associated genes. Importantly, these phenotypes emerged only when ANO1 deletion occurred during development, whereas adult-stage manipulation failed to reproduce them, underscoring a critical developmental window for habenular–thalamocortical circuit maturation. This developmental specificity represents a central novelty of the model and provides new insight into how early-life dysregulation of habenular cholinergic signaling contributes to schizophrenia-related pathophysiology.

**Supplementary Information:**

The online version contains supplementary material available at 10.1186/s13041-025-01266-y.

## Introduction

Schizophrenia is a chronic and severe psychiatric disorder marked by profound disruptions in perception, cognition, and effect, and affect, typically emerging during late adolescence or early adulthood [1]. However, a subset of patients experiences early-onset schizophrenia (EOS), with symptoms manifesting during childhood or adolescence. EOS is often associated with a more severe clinical course, greater cognitive impairment, and poorer long-term outcomes, suggesting a progressively deteriorating neurodevelopmental trajectory [1]. Understanding the early pathophysiological mechanisms underlying EOS is therefore critical for improving early diagnosis and developing effective therapeutic strategies. Animal models have proven essential in investigating the neurobiological basis of schizophrenia, providing controlled system to dissect the contributions of genetic, developmental, and environmental risk factors [2, 3]. In particular, models mimicking early-onset disease are valuable tools for exploring how disruptions during critical periods of brain development contribute to the onset and progression of schizophrenia. For example, neurodevelopmental models such as the neonatal ventral hippocampal lesion (NVHL) model have demonstrated that early brain insults can lead to delayed behavioral and neurochemical abnormalities characteristic of schizophrenia, including cognitive dysfunction and abnormal stress responses [1, 4].

A central feature of schizophrenia is dysregulation of dopaminergic signaling. The dopamine hypothesis has evolved to suggest that hyperdopaminergic activity in subcortical regions, particularly the striatum, contributes to positive symptoms, while hypodopaminergic states in the prefrontal cortex are linked to cognitive and negative symptoms [5, 6]. Animal studies have shown that early neurodevelopmental insults can induce persistent alteration in dopaminergic circuits, highlighting the importance of early-onset models in elucidating such vulnerabilities [7–9]. In addition to dopaminergic dysfunction, abnormal neural oscillations, particularly in the gamma frequency band, have been identified as electrophysiological biomarkers of schizophrenia. These reflect disrupted cortical network synchrony and implicate both glutamatergic and dopaminergic systems [10, 11]. Furthermore, auditory processing deficits – a well-documented symptom domain in schizophrenia – are closely associated with hyperactivity of mesolimbic dopaminergic pathways, a mechanism thought to underlie auditory hallucinations. This hyperdopaminergic state may be exacerbated by hilar dysfunction, which disrupts hippocampal-thalamocortical connectivity. Aberrant thalamocortical coupling - particularly involving the hippocampus - may impair the brain’s ability to distinguish internally generated from externally derived auditory information, thereby contributing to psychotic symptoms such as hallucination [6, 12, 13]. Such electrophysiological and circuit-level alteration have been successfully replicated in rodent models, providing valuable translational relevance for understanding disease mechanisms and testing potential interventions [14, 15].

Despite these advances, modeling schizophrenia in animals remains inherently challenging, as no single model can fully recapitulate the disorder’s multifaceted clinical and neurobiological features [3, 14]. Interspecies differences in brain structure and function further complicate the translation of preclinical findings to human disease [2]. Therefore, careful validation of animal models - particularly those reflect development timing and dopaminergic dysfunction - is crucial to bridge the gap between basic research and clinical application.

In this study, we propose a novel early-onset schizophrenia mouse model based on early hilar dysfunction, which induces dopaminergic alterations in auditory circuits implicated in schizophrenia. Moreover, our findings suggest that hilar dysfunction does not significantly affect dopaminergic signaling in adulthood, indicating the existence of a critical developmental window during which hilar pathology exerts its pathogenic effects.

## Materials and methods

### Animals

Male ANO1^f/f^ mice on a C57BL/6 background, aged 4–17 weeks, were used for all experiments. To achieve cholinergic neuron-specific deletion of ANO1, ANO1^f/f^ mice were crossed with ChAT-Cre transgenic mice (B6;129S6-Chat^tm2(cre)Lowl^/J, Jackson Laboratory, #006410). All animal procedures were conducted in accordance with the guidelines and regulation approved by the Institution Animal Care and Use Committee (IACUC) of the Korea Institute of Science and Technology (KIST, Seoul, Korea).

### RT-PCR and real-time PCR

Total RNA was isolated from various mouse brain regions, including the medial habenula, hippocampus, striatum, cortex, and ventral medial geniculate nucleus (MGv) using RNA purification kit (#305 − 101, GeneAll, Seoul, Korea), following the manufacturer’s instructions. cDNA was synthesized from 500ng of total RNA using the SensiFAST™ cDNA synthesis kit (#BIO-65053, Bioline, London, UK). Quantitative real-time PCR (qPCR) was performed using the SensiFAST™ probe Hi-ROX kit (#BIO-82205, Bioline). The following qPCR Assay primers and probes (Integrated DNA Technologies; IDT, Coralville, IA, USA) were used [16]:

ANO1: Forward: 5′-GTGGAGTCATACTGGAACATGTAG‐3′, Reverse: 5′‐GCCAGAGTCTTAGAGAAGTCAC‐3′, Probes: Probe − 5′‐/56‐FAM/AGAATCCAG/ZEN/AAACAAAGAGACCGACAAGG/3IABkFQ/‐3′, GAPDH: Forward: 5′‐GTGGAGTCATACTGGAACATGTAG‐3′, Reverse: 5′‐AATGGTGAAGGTCGGTGTG‐3′, Probes: 5′‐/56‐FAM/TGCAAATGG/ZEN/CAGCCCTGGTG/3IABkFQ/‐3′. Relative gene expression levels were calculated using the 2^−ΔΔCt^ method, with GAPDH as the internal control. All qPCR experiments were performed in triplicate. For conventional reverse transcription PCR (RT-PCR), cDNA was amplified using 2XTOPsimple™ DyeMIX -Tenuto (Enzynomics, Daejeon, Korea). The following primer pairs were used: Drd2: Forward: 5′-GGATGTCATGATGTGCACAGC-3′, Reverse: 5′-CTTGCGGAGAACGATG-3′, GAPDH: Forward: 5′-GTCTTCACCACCATGGAGAA-3′, Reverse: 5′-GCATGGACTGTGGTCATGAG-3′. Drd2 mRNA expression levels were normalized to GAPDH. All RT-PCR experiments were independently repeated at least three times.

### Immunohistochemistry

Brains from 8-week-old ANO1^f/f^ and ANO1 cKO mice were collected following transcardial perfusion with ice-cold saline, immediately followed by fixation with 4% paraformaldehyde (PFA; Sigma-Aldrich, St. Louis, MO, USA) in PBS. The brains were post-fixed overnight at 4 °C, then coronally sectioned at a thickness of 40 μm using a vibratome (#VT1200s, Leica Microsystems, Wtzlar, Germany). Sections were stored in PBS until further processing. For antigen retrieval, free-floating sections containing the ventral medial geniculate nucleus (mGv) were incubated in 10 mM sodium citrate buffer (pH 6.0) at 80 °C for 30 min using a water bath. Following retrieval, tissues were permeabilized in 0.5% Triton X-100 in PBS for 40 min at room temperature. Sections were then blocked for 1 h in a blocking solution containing 3% bovine serum albumin (BSA) and 5% donkey serum in 0.3% Triton X-100 in PBS to prevent nonspecific binding. Blocked sections were incubated overnight at 4 °C with the following primary antibodies, diluted 1:300 in blocking solution: anti-S100β (#ab868, Abcam, Cambridge, UK), anti-D2 dopamine receptor (D2DR; B-10, #sc-5303, Santa Cruz Biotechnology, Dallas, TX, USA), and anti-MAP2 (#ab5392, Abcam). The next day, sections were washed and incubated for 1 h at room temperature with Alexa Fluor-conjugated secondary antibodies (488, 594, or 647; 1:300, Jackson ImmunoResearch Laboratories, Inc., West Grove, PA, USA), also diluted in blocking solution. After secondary incubation, nuclei were counterstained with DAPI (1:1000, Sigma) for 15 min at room temperature. Finally, sections were mounted onto glass slides using DAKO fluorescence mounting medium (#S3023, Agilent Technologies, Santa Clara, CA, USA), and images were acquired with a Nikon A1 confocal laser-scanning microscope.

### Stereotaxic injection

Eight-week-old mice were deeply anesthetized with tribromoethanol (Avertin; 2,2,2,-tribromoethanol in 2-methyl-2-butanol) administered via intraperitoneal injection and positioned in a stereotaxic apparatus (Kopf Instruments, Tujunga, CA, USA). The scalp was incised, and bilateral craniotomies were made at the following stereotaxic coordinates relative to bregma: anteroposterior (AP), -1.8 mm; mediolateral (ML), ± 0.5 mm. AAV-ANO1-shRNA-mCherry or control AAV-Scrambled-shRNA-mCherry was bilaterally injected into the medial habenula (mHb) at a depth of 2.7 mm dorsoventral (DV) from the dura using a glass microdispenser (Hamilton company, Reno, NV, USA) connected to a syringe pump (KD Scientific, Holliston, MA, USA). Viral solutions were infused at a constant rate of 0.2 µl/min. The microdispenser was left in place for 2 min before and after injection to minimize reflux along the needle tract. Successful targeting of the mHb was verified two weeks post-injection by visualizing mCherry fluorescence expressed from the AAV vector.

### DREADD-mediated mHb Inhibition with CNO

AAV-hsyn-DIO-hM4Di (Gi)-mCherry (#44362 serotype 2, Addgene, Watertown, MA, USA) was stereotaxically injected into the mHb of adult male ChAT-Cre mice (10 weeks old) under anesthesia. Following viral injection, mice were allowed to recover for five weeks to ensure sufficient expression of hM4Di. For behavioral experiments, Clozapine-N-Oxide (CNO; #C0832, Sigma-Aldrich) was administered intraperitoneally at a dose of 0.5 mg/kg, diluted in artificial cerebrospinal fluid (ACSF) to a final concentration of 10 µM. Pre-pulse inhibition (PPI) testing was conducted 30 min after CNO injection to assess sensorimotor gating. Two weeks after behavioral testing, mice were sacrificed and brain tissues were collected for RT-PCR analysis to verify gene expression changes associated with chemogenetic manipulation.

### Pre-pulse inhibition (PPI)

The pre-pulse inhibition (PPI) test was used to assess sensorimotor gating, which reflects the animal’s ability to suppress a startle response when a strong acoustic stimulus (pulse) is preceded by a weaker, non-startling stimulus (pre-pulse). All tests were conducted in sound-attenuating isolation chambers designed for startle response measurements. During the test, mice were gently restrained in an isolation chamber, with a constant background noise set at 65 dB. Each session consisted of five trial types: (1) Pulse-alone trials (P): a single burst of white noise at 120 dB for 40ms; (2–4) Pre-pulse + pulse trials (PP74P, PP82P, PP90P): a 20 ms pre-pulse at 74, 82, or 90 dB, following by a 120 dB pulse delivered 100 ms after the onset of the pre-pulse; (5) No-stimulus (NS) trials, in which only the background noise was presented. Each session began with a 10-min acclimation period under background noise, followed by five initial P trials. The main testing phase included ten blocks, each containing all five trial types (P, PP74P, PP82P, PP90P, and NS) presented in pseudorandom order, and ended with another five P trials. Inter-trial intervals varied randomly between 12 and 30 s to prevent anticipatory responses. The degree of PPI was calculated as the percentage reduction in startle amplitude during the pre-pulse + pulse trials relative to the pulse-alone trials, using the following formula:

$$ \begin{gathered} \% PPI \hfill \\ = \left[ {1 - \frac{{\left( {startle\:amplitude\:on\:pre - pulse + pulse\:trial} \right)}}{{\left( {startle\:amplitude\:on\:pulse - alone\:trial} \right)}}} \right] \times 100\% \hfill \\ \end{gathered} $$

Startle amplitude was defined as the average response across all P trials, excluding the first and last five trials to minimize habituation and sensitization effects.

### Haloperidol treatment

Haloperidol (#H1512, Sigma-Aldrich) was dissolved in 0.3 M HCl in saline, and the pH of the solution was adjusted to 5.5–6.0 using sodium hydroxide. For behavioral experiments, including pre-pulse inhibition (PPI) and cocaine-induced locomotor activity, haloperidol was administered intraperitoneally at a dose of 0.1 mg/kg, 30 min prior to testing to allow sufficient time for drug onset [17].

### Cocaine-induced locomotor activity test

Locomotor activity was assessed using the open field test (OFT) to evaluate behavioral response to cocaine administration. Mice were individually placed in an open field box (40 × 40 cm) and allowed to explore freely. Locomotion was recorded for a total of 60 min, consisting of two consecutive 30-min phases. Prior to testing, animals received an intraperitoneal (*i.p.*) injection of either vehicle or haloperidol (0.1 mg/kg). Because the behavioral effect of haloperidol typically emerges within 30 min, cocaine (15 mg/kg, *i.p.*) or saline was administered exactly 30 min after the initial injection. Locomotor activity was recorded continuously before and after the cocaine injection to capture both baseline activity and drug-induced hyperlocomotion [18]. All animals were habituated to handling and mock injections for three consecutive days prior to testing to minimize novelty-induced hyperactivity. Locomotor activity was quantified as total distance traveled (in cm) using EthoVision XT 11.5 software (Noldus Information Technology, Leesburg, VA, USA). The testing chamber were thoroughly cleaned with 70% ethanol followed by distilled water between trials to eliminate olfactory cues.

### Laser capture microdissection (LCM)

Brain tissues were embedded in the optimum cutting temperature compound (O.C.T.) Compound (Scigen, Paramount, CA, USA) and stored at -80 °C until use. Coronal Sect. (10 μm thick) were prepared through the habenula region using a cryostat (#CM1860UV, Leica). Sections were mounted on glass slide and air-dried for 5 min. The sections were then dehydrated and fixed by sequential immersion in 50%, 75%, 95%, and 100% ethanol for 30 s each, followed by xylene for 1 min. After dehydration, the medial habenula (mHb) region was identified under a microscope and isolated using laser capture microdissection (LCM) software. The captured mHb regions were collected in a single adhesive cap and immediately processed for RNA isolation [19, 20].

### RNA isolation

Total RNA was extracted from laser-captured mHb tissue using TRIzol reagent (#15596026, Invitrogen) according to the manufacturer’s instructions. RNA integrity was assessed using the Agilent 2100 Bioanalyzer with the RNA 6000 Nano Chip (Agilent Technologies, Amstelveen, Netherlands), and RNA concentration was measured using the ND-2000 Spectrophotometer (Thermo Fisher Scientific, Waltham, MA, USA).

### Library preparation and sequencing

For both control and experimental samples, libraries were prepared using the QuantSeq 3’ mRNA-Seq Library Prep Kit (Lexogen GmbH, Vienna, Austria), following the manufacturer’s instructions. Briefly, 500ng of total RNA was used per sample. An oligo-dT primer containing an Illumina-compatible adapter sequence at its 5’ end was hybridized to the poly(A) tail of mRNA, followed by reverse transcription. After degradation of the RNA template, second-strand synthesis was initiated using a random primer harboring a 5’ Illumina-compatible sequence. Double-stranded cDNA libraries were purified using magnetic beads to remove residual enzymes and reaction components. The libraries were then PCR-amplified to incorporate complete Illumina adapter sequences required for cluster generation. Final libraries were purified and quality-checked before sequencing. High-throughput sequencing was performed on the NextSeq 500 platform (Illumina, Inc., San Diego, CA, USA) with single-end 75 bp reads.

### RNA-Seq data analysis

QuantSeq 3’ mRNA-Seq reads were aligned to the reference genome or transcriptome using Bowtie2 [21]. Bowtie2 indices were generated from either the genome assembly or representative transcript sequences. Aligned reads were processed to assemble transcripts, estimate expression levels, and identify differentially expressed genes (DEGs). Read counts were obtained using Bedtools [22] based on both uniquely and multiply mapped reads. Normalization of read count data was performed using the quantile normalization method implemented in the edgeR package [23]. Gene ontology and functional classification analyses were conducted using DAVID (http://david.abcc.ncifcrf.gov/) and annotations were verified through NCBI Medline databases (http://www.ncbi.nlm.nih.gov/).

### Statistical analysis

All statistical analyses were conducted using GraphPad Prism software (version 10; GraphPad Software, San Diego, USA). Data were presented as the mean ± standard error of the mean (SEM) from at least three independent experiments. Statistical significance was assessed using one-way analysis of variance (ANOVA) followed by Tukey’s post-hoc test for multiple comparisons. Differences were considered statistically significant at *p* < 0.05.

## Results

### c-fos expression is significantly reduced in the mPFC of ANO1 cKO mice

Alterations in medial prefrontal cortex (mPFC) activity have been consistently reported in patients with schizophrenia and are thought to contribute to cognitive deficits and negative symptoms associated with the disorder [1, 2, 10]. To investigate whether ANO1 conditional knockout (cKO) mice exhibit similar changes in mPFC neuronal activity, we performed c-Fos immunostaining, a widely used marker of neuronal activation.

**Fig. 1:**
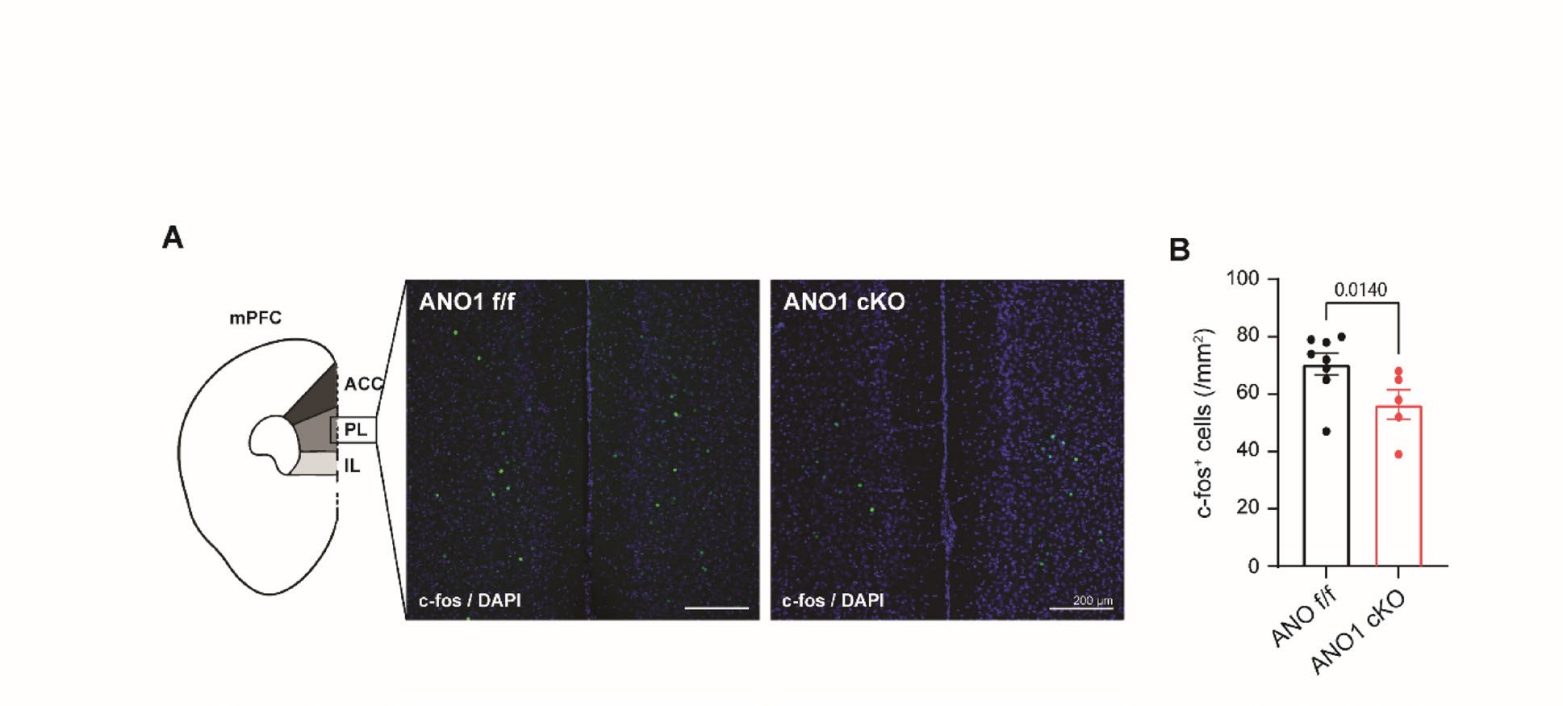
Reduced mPFC neuronal activity in ANO1 cKO mice. **A** Schematic illustration of the medial prefrontal cortex (mPFC) regions analyzed, including the anterior cingulate cortex (ACC), prelimbic (PL), and infralimbic (IL) areas (left). Representative images of c-Fos immunostaining (green) with DAPI nuclear counterstaining (blue) in the mPFC of ANO1^f/f^ (control) and ANO1 cKO mice (right). Scale bar = 200 μm. **B** Quantification of c-Fos–positive cells in the mPFC. Data are presented as mean ± SEM. The exact p-values ​​are shown in the figure

Although we were unable to investigate other brain regions extensively, robust c-Fos expression was observed throughout the mPFC, including the anterior cingulate cortex (ACC), prelimbic (PL), and infralimbic (IL) regions, in control ANO1^f/f^ mice. In contrast, ANO1 cKO mice exhibited a marked reduction in c-Fos–positive cells in the mPFC (Fig. 1A). Quantitative analysis revealed that the number of c-Fos–positive cells was significantly decreased in the mPFC of ANO1 cKO mice compared to controls (unpaired t-test) (Fig. 1B). These results suggest reduced neuronal activity in the mPFC of ANO1 cKO mice, consistent with patterns observed in schizophrenia.

### ANO1 cKO mice show reduced PPI and heightened cocaine-induced hyperactivity, both of which are restored by haloperidol

**Fig. 2:**
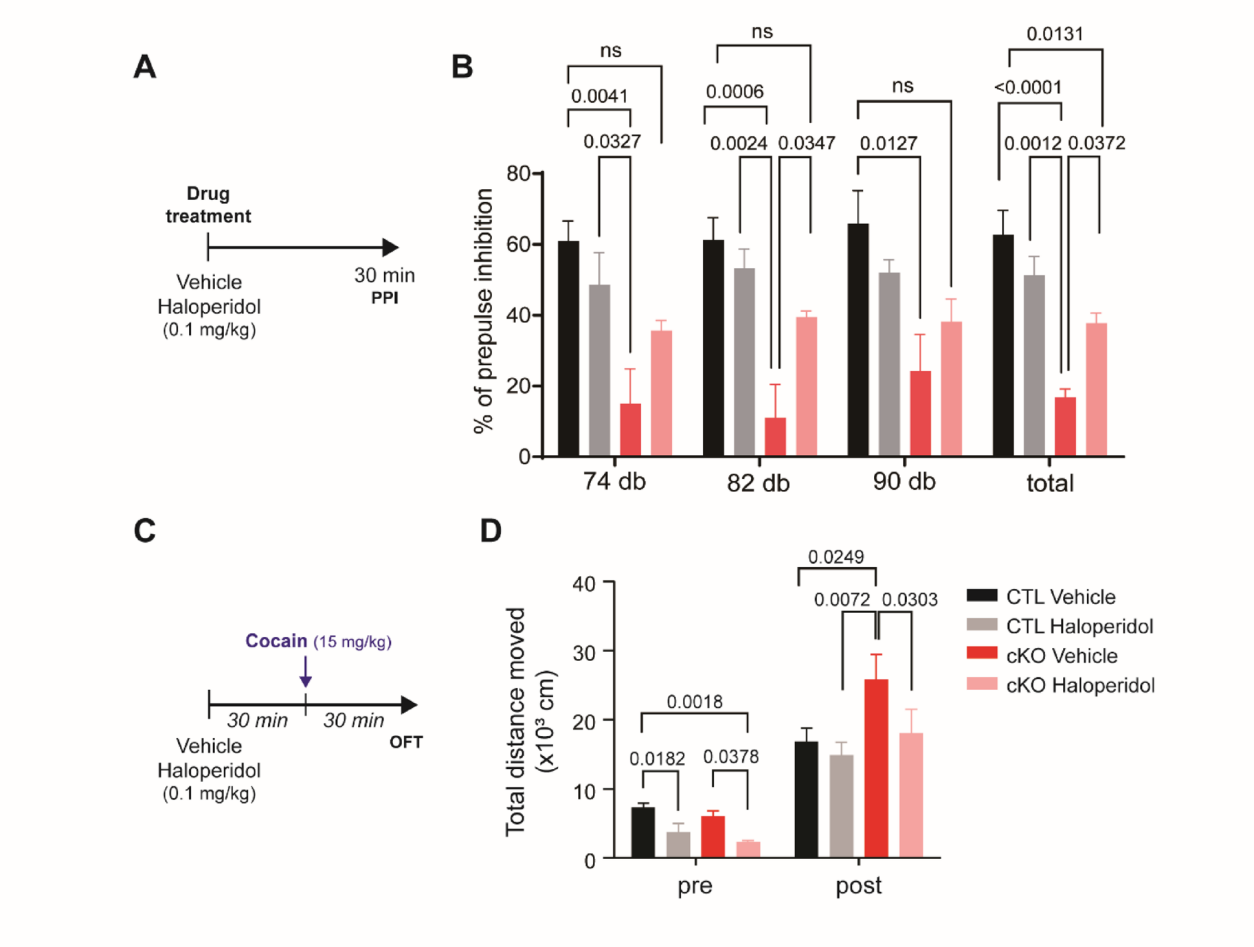
Haloperidol reverses sensorimotor gating deficits and psychostimulant-induced hyperactivity in ANO1 cKO mice. **A** Experimental timeline for PPI testing following vehicle or haloperidol (0.1 mg/kg, i.p.) administration. **B** Percentage of prepulse inhibition (%PPI) in control (CTL) and ANO1 cKO mice across three prepulse intensities (74 dB, 82 dB, 90 dB) and overall average (total). ANO1 cKO mice exhibited significantly reduced PPI, which was rescued by haloperidol treatment. **C** Experimental timeline for open field testing (OFT) following cocaine (15 mg/kg, i.p.) administration, with or without haloperidol pre-treatment. **D** Total distance moved in the OFT before (pre) and after (post) cocaine injection. ANO1 cKO mice displayed enhanced locomotor activity following cocaine, which was significantly reduced by haloperidol. Data are presented as mean ± SEM. The exact p-values ​​are shown in the figure

To assess sensorimotor gating deficits, we performed pre-pulse inhibition (PPI) testing in ANO1 cKO mice, as impairments in PPI are commonly observed in schizophrenia models. ANO1 cKO mice exhibited significantly reduced PPI compared to control mice across pre-pulse intensities of 74 dB, 82 dB, and 90 dB, as well as in overall PPI levels (Fig. 2A, B). Notably, administration of haloperidol (0.1 mg/kg, i.p.) significantly restored PPI performance in ANO1 cKO mice to levels comparable to controls, indicating that dopaminergic modulation can rescue the PPI deficits observed in the cKO mice.

In addition, we evaluated the locomotor response to cocaine, as hyper-responsivity to psychostimulants is another hallmark of dopaminergic dysregulation in schizophrenia models. Following cocaine administration (15 mg/kg, i.p.), ANO1 cKO mice displayed significantly increased locomotor activity compared to controls (Fig. 2C, D). Pre-treatment with haloperidol significantly attenuated cocaine-induced hyperactivity in both control and cKO mice. These results indicate that ANO1 cKO mice exhibit schizophrenia-relevant behavioral phenotypes, including sensorimotor gating deficits and heightened sensitivity to psychostimulant-induced hyperactivity, both of which can be ameliorated by antipsychotic intervention.

### DRD2 expression increases in the MGv of ANO1 cKO mice from 4 weeks of age

**Fig. 3:**
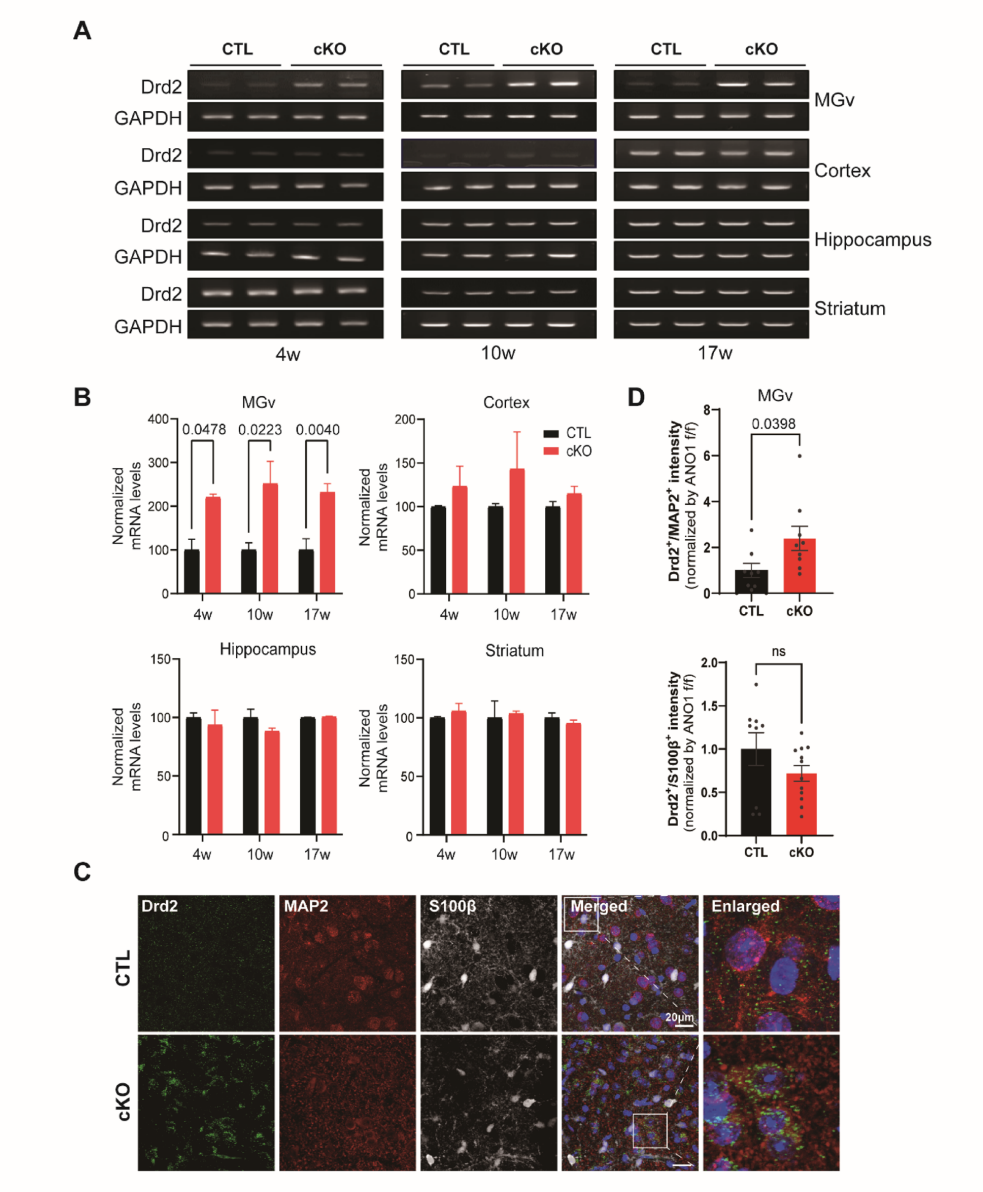
Upregulation of Drd2 expression in the MGv of ANO1 cKO mice. **A** Representative RT-PCR bands showing Drd2 and GAPDH expression in the MGv, cortex, hippocampus, and striatum of control (CTL) and ANO1 cKO mice at 4, 10, and 17 weeks of age. **B** Quantification of Drd2 mRNA levels normalized to GAPDH in each brain region. Significant increases in Drd2 mRNA were observed in the MGv of cKO mice across all ages examined, while no significant changes were detected in the cortex, hippocampus, or striatum. Data are presented as mean ± SEM. **C** Immunofluorescence staining of Drd2 (green), neuronal marker MAP2 (red), and astrocytic marker S100β (white) in the MGv of CTL and ANO1 cKO mice. Merged and enlarged views illustrate co-localization patterns. Scale bar = 20 μm. **D** Quantification of Drd2 fluorescence intensity normalized to ANO1^f/f^ levels in MAP2-positive and S100β-positive cells. Drd2 signal was significantly elevated in MAP2-positive neurons of cKO mice, while no significant change was observed in S100β-positive astrocytes. Data are presented as mean ± SEM. The exact p-values ​​are shown in the figure

To examine potential alterations in dopaminergic signaling associated with ANO1 deficiency, we assessed Drd2 mRNA expression levels in various brain regions of ANO1 conditional knockout (cKO) mice across developmental stages. RT-PCR analyses revealed that Drd2 expression was significantly increased in the ventral medial geniculate nucleus (MGv) of ANO1 cKO mice as early as 4 weeks of age and remained elevated at 10 and 17 weeks (Fig. 3A, B). In contrast, no significant differences in Drd2 expression were observed between cKO and control mice in the cortex, hippocampus, or striatum at any examined time point.

To further determine in which cell types Drd2 expression was altered, and to verify these changes at the protein level, we performed immunofluorescence staining for Drd2 together with neuronal (MAP2) and astrocytic (S100β) markers. Quantitative analysis demonstrated that the increased Drd2 signal in cKO mice was predominantly localized to MAP2-positive neurons, while Drd2 levels in S100β-positive astrocytes were not significantly different between genotypes (Fig. 3C, D). These results indicate a neuron-specific upregulation of Drd2 protein expression in the MGv of ANO1 cKO mice, suggesting dopaminergic alterations that may contribute to schizophrenia-related pathophysiology. To assess whether similar changes could be induced by acute manipulation of medial habenula (mHb) activity in adulthood, we performed shRNA-mediated ANO1 knockdown and chemogenetic inhibition experiments. Adult ANO1^f/f^ mice received bilateral injections of pAAV-ANO1 shRNA-mCherry or scrambled shRNA-mCherry as a control into the mHb. RT-PCR analysis at 10 weeks revealed no significant alterations in Drd2 expression in either the MGv or striatum between ANO1 shRNA-treated and scrambled shRNA-treated mice (Fig. S1A–C). In a parallel approach, adult Chat-Cre mice were injected with pAAV-hM4Di-mCherry into the mHb. Activation of hM4Di receptors with clozapine-N-oxide (CNO) did not significantly affect prepulse inhibition (PPI) performance compared to saline-treated controls (Fig. S1D–F). Moreover, RT-PCR analyses performed at 17 weeks showed no significant changes in Drd2 mRNA levels in the MGv, cortex, hippocampus, or striatum following chemogenetic inhibition of the mHb (Fig. S1G, H).

Collectively, these findings indicate that Drd2 upregulation in the MGv is specific to developmental ANO1 deficiency and cannot be replicated by acute suppression of mHb activity in adulthood. This suggests a critical developmental window during which ANO1 loss impacts dopaminergic circuitry relevant to schizophrenia-related phenotypes.

### Transcriptomic alterations in the mHb of ANO1 cKO mice reveal schizophrenia-related signatures not recapitulated by acute ANO1 suppression

**Fig. 4:**
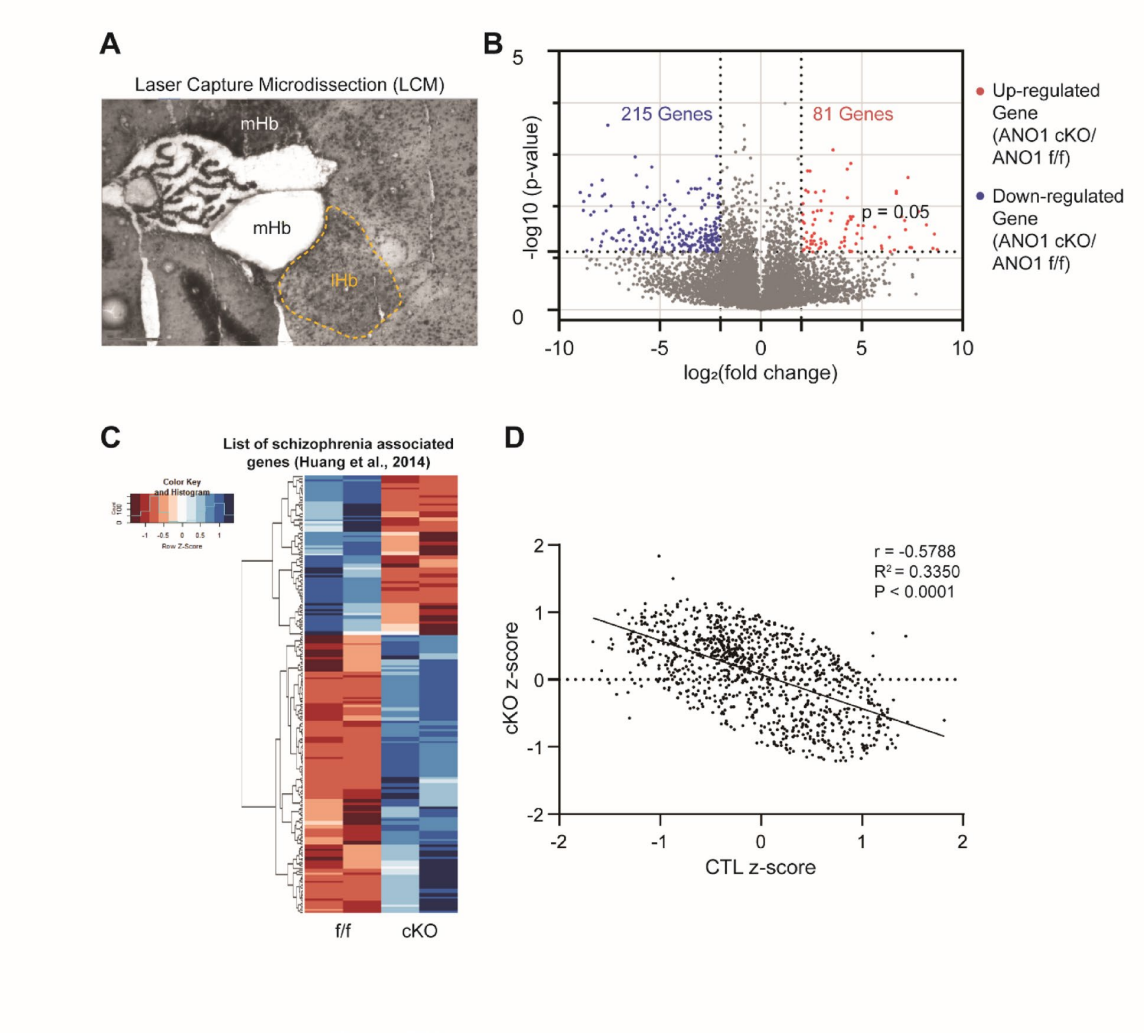
Transcriptomic alterations in the mHb of ANO1 cKO mice. **A** Representative image of laser capture microdissection (LCM) used to isolate the medial habenula (mHb) region from mouse brain sections. **B** Volcano plot depicting differentially expressed genes between ANO1 cKO and f/f mice. Red dots indicate significantly upregulated genes (*n* = 81), and blue dots indicate significantly downregulated genes (*n* = 215) in cKO mice relative to controls. **C** Heatmap showing expression profiles of schizophrenia-associated genes (Huang et al., 2014) in ANO1^f/f^ and cKO mice. Each row represents a gene, and colors indicate relative expression levels (red = high; blue = low). **D** Scatter plot demonstrating a significant negative correlation between z-scores of gene expression in ANO1^f/f^ and cKO mice. *r* = − 0.5788, R² = 0.3350. The exact p-values ​​are shown in the figure

To investigate transcriptomic changes associated with ANO1 deficiency in the medial habenula (mHb), we performed RNA sequencing on laser-capture microdissected mHb tissue from ANO1^f/f^ and cKO mice (Fig. 4A). Differential gene expression analysis identified 81 significantly upregulated and 215 significantly downregulated genes in ANO1 cKO mice relative to controls (Fig. 4B). Comparison of these differentially expressed genes with a curated list of schizophrenia-associated genes [24] revealed that the overlapping 217 genes, as well as the ANO1 and GPR151 genes, exhibited marked differences in expression patterns in ANO1 cKO mice (Fig. 4C, Table S1). Correlation analysis demonstrated a significant negative relationship between gene expression z-scores in ANO1 cKO and control mice (*r* = − 0.5788, R² = 0.3350, *p* < 0.0001), suggesting that ANO1 deficiency induces transcriptomic alterations inversely related to typical expression profiles in schizophrenia (Fig. 4D). These results indicate that developmental ANO1 loss leads to widespread molecular changes involving schizophrenia-related genes.

To determine whether similar transcriptomic alterations could be induced by acute reduction of ANO1 expression in adulthood, we performed ANO1 shRNA-mediated knockdown in the mHb of adult mice. RT-PCR analyses revealed significant reductions in ANO1 and c-fos mRNA levels, confirming effective knockdown and reduced neuronal activity. However, expression of GPR151, a gene associated with habenular function, was unchanged, suggesting that the knockdown did not globally affect mHb gene expression (Fig. S2A). Further analysis of differentially expressed transcripts following ANO1 shRNA treatment revealed enrichment in Gene Ontology (GO) terms related to synaptic function, protein transport, transcriptional regulation, and cellular stress responses (Fig. S2B). However, these changes were modest and did not involve broad dysregulation of schizophrenia-associated genes, in contrast to the pronounced transcriptomic alterations observed in developmental ANO1 cKO mice. These findings suggest that developmental timing of ANO1 deficiency is critical for inducing schizophrenia-related molecular signatures in the mHb.

## Discussion

In this study, we demonstrate that developmental loss of ANO1 function in cholinergic neurons of the medial habenula (mHb) leads to behavioral and molecular alterations relevant to schizophrenia pathophysiology. Mice lacking ANO1 in these neurons exhibited diminished medial prefrontal cortex (mPFC) activity, deficits in prepulse inhibition (PPI), and heightened susceptibility to psychostimulant-induced hyperactivity. These phenotypes are highly reminiscent of neurophysiological and behavioral endophenotypes frequently reported in individuals with schizophrenia [1, 10]. Importantly, treatment with haloperidol, a typical antipsychotic agent and dopamine D2 receptor antagonist, successfully reversed these behavioral deficits, suggesting a crucial role of dopaminergic dysfunction in mediating these abnormalities.

A particularly notable observation in our study is the selective upregulation of Drd2 mRNA expression in the ventral medial geniculate nucleus (MGv) of ANO1 cKO mice, primarily localized to MAP2-positive neurons, indicative of neuronal specificity. The MGv functions as a key thalamic relay transmitting auditory information to the auditory cortex. This raises compelling questions about the involvement of thalamocortical pathways in the emergence of schizophrenia-related symptoms. Previous human neuroimaging and post-mortem studies, as well as animal models, have consistently reported that disruptions in thalamic projections to the auditory cortex represent a prominent pathological feature in schizophrenia [2, 21]. Such thalamocortical dysfunctions have been proposed to contribute to impaired sensory gating, abnormal auditory processing, and the development of perceptual disturbances such as auditory hallucinations, a core symptom of schizophrenia.

Our data suggest that developmental ANO1 deficiency may trigger molecular and potentially functional remodeling within the MGv, leading to altered thalamocortical signaling. The observed increase in Drd2 expression could potentiate dopaminergic modulation of thalamic neuronal activity, potentially disturbing the fidelity of auditory information processing en route to cortical regions. This hypothesis aligns well with previous findings that implicate hyperdopaminergic states in thalamic circuits as contributors to sensory misperceptions and hallucinations in schizophrenia [2, 25]. However, the precise functional consequences of elevated Drd2 signaling in the MGv remain to be elucidated. Moreover, although circuit-level analyses linking ANO1 deficiency in the mHb to Drd2 upregulation in the MGv were not performed in this study, indirect modulation through the mHb–interpeduncular nucleus (IPN)–raphe pathway has been reported to influence both dopaminergic tone and thalamocortical signaling [26]. Given that Drd2 overexpression in the MGv can hypersensitize the MGv–auditory cortex pathway, increased Drd2 activity may disrupt excitatory relay fidelity and lead to abnormal sensory filtering or hallucination-like percepts. Taken together, these findings suggest that developmental disturbances in mHb cholinergic signaling could influence MGv–auditory cortical activity through midbrain dopaminergic circuits, ultimately resulting in sensory gating alterations and thalamocortical dysregulation associated with schizophrenia-related phenotypes.

Beyond regional dopaminergic changes, transcriptomic profiling revealed widespread gene expression alterations in the mHb of ANO1 cKO mice. These changes included significant dysregulation of numerous genes previously implicated in schizophrenia pathophysiology [22]. Strikingly, neither acute ANO1 knockdown via shRNA nor chemogenetic inhibition of mHb neurons in adult mice replicated these transcriptomic alterations or induced similar behavioral abnormalities. This highlights the critical importance of developmental timing, supporting the neurodevelopmental hypothesis of schizophrenia. According to this model, early-life disruptions in brain maturation can set the stage for long-lasting molecular, cellular, and circuit-level changes that remain latent until adolescence or early adulthood, when they may manifest as psychiatric symptoms [1, 27]. Our findings thus underscore the notion that transient or acute perturbations in adulthood may be insufficient to recapitulate the complex molecular landscape and behavioral phenotypes associated with developmental disruptions.

Despite the strength of our findings, certain limitations must be acknowledged. Although we identified elevated Drd2 expression in the MGv, the downstream impact on auditory cortical physiology and auditory perception remains to be directly demonstrated. Additionally, while haloperidol successfully mitigated behavioral impairments, its effects on the broader transcriptomic landscape induced by developmental ANO1 deficiency have yet to be elucidated. It remains unclear whether antipsychotic treatment could also reverse the molecular changes or restore normal thalamocortical circuitry in this model. Future studies aimed at dissecting these mechanistic links will be critical to translating these findings into therapeutic insights can normalize molecular changes and thalamocortical circuit dysfunctions in this model.

## Conclusion

Taken together, our study provides novel evidence that developmental ANO1 deficiency in mHb cholinergic neurons induces schizophrenia-relevant behavioral and molecular phenotypes. These alterations appear to involve dysregulation of thalamocortical pathways, including increased Drd2 expression within the MGv, which may compromise auditory information processing and contribute to symptomatology such as hallucinations. Our data highlight the significance of early developmental disruptions in shaping neural circuits and molecular profiles implicated in schizophrenia, and suggest that ANO1 may represent a potential molecular target for understanding disease mechanisms and developing innovative therapeutic strategies. Further research is warranted to clarify the causal relationships between ANO1 deficiency, thalamocortical dysfunction, and schizophrenia-like behaviors, and to explore whether pharmacological interventions can effectively normalize these alterations.

## Supplementary Information

Below is the link to the electronic supplementary material.

Supplementary Material 1

Supplementary Material 2

Supplementary Material 3

## Data Availability

All data supporting the findings of this study are available within the main manuscript and its supplementary information files.
