# Supplementary figures and images for: TMEM16A ablation in cholinergic medial habenula neurons induces early-onset schizophrenia-like phenotypes in mice
Source: Mol Brain. 2026 Jan 12;19:5. doi: 10.1186/s13041-025-01266-y (PMC12797405; doi:10.1186/s13041-025-01266-y)

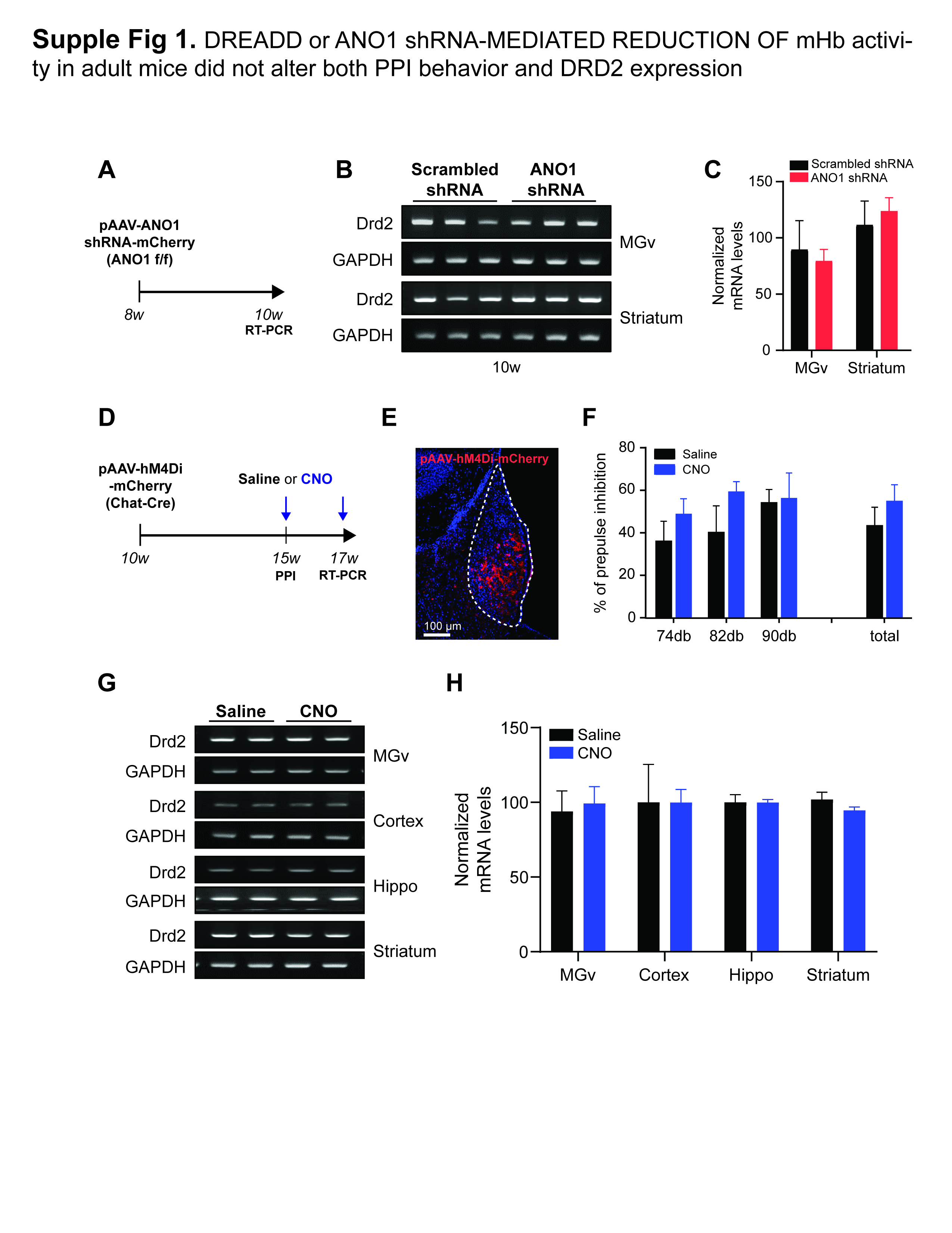

Supplement: Supplementary file 2 — Supplementary Material 2 [file 13041_2025_1266_MOESM2_ESM.tif]

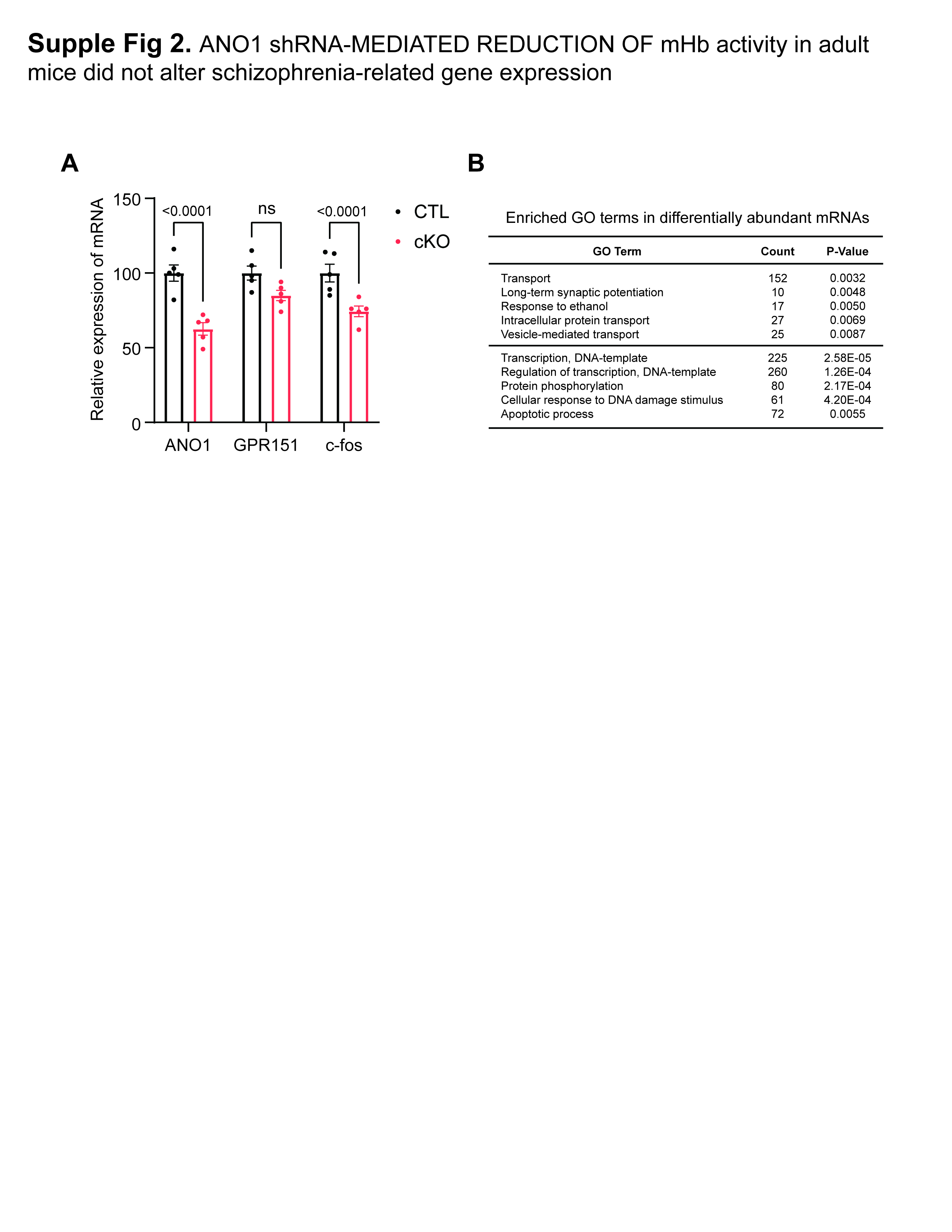

Supplement: Supplementary file 3 — Supplementary Material 3 [file 13041_2025_1266_MOESM3_ESM.tif]
